# Effectiveness, Acceptability and Safety of Pharmaceutical Management for Combat-Related PTSD in Adults Based on Systematic Review of Twenty-Two Randomized Controlled Trials

**DOI:** 10.3389/fphar.2021.805354

**Published:** 2022-01-18

**Authors:** Jin-Zhu Yan, Jia-Ling Liu, Xiao-Zheng Li, Zhi-Xin Zhang, Run-Ben Liu, Chao Zhang, Qin-Qin Gong

**Affiliations:** ^1^ Department of Obstetrics, Taihe Hospital, Hubei University of Medicine, Shiyan, China; ^2^ Center for Evidence-Based Medicine and Clinical Research, Taihe Hospital, Hubei University of Medicine, Shiyan, China; ^3^ Center of Women’s Health Sciences, Taihe Hospital, Hubei University of Medicine, Shiyan, China

**Keywords:** post-traumatic stress disorder, veterans, pharmacological interventions, depression, anxiety

## Abstract

**Objective:** This study assessed the efficacy, acceptability, and safety of pharmaceutical management for combat-related post-traumatic stress disorder (PTSD) to provide a clinical decision-making basis for clinicians.

**Method:** A comprehensive search was conducted using Ovid MEDLINE, Ovid EMBASE, Cochrane Library, Scopus, ScienceDirect, and Web of Science for randomized controlled trails (RCTs), which reported pharmaceutical management and placobo for adults with combat-related PTSD, that were published until April 21, 2021. The effectiveness, acceptability, and adverse events (AEs), were designed as interested outcomes. The change in total symptoms of combat-related PTSD according to the clinician rating scale was defined as primary outcome, and the others were defined as secondary outcomes.

**Results:** Twenty-two RCTs with 1,221 patients were involved. Compared with placebo, overall active comparators had statistical differences for all outcomes, including the change in total symptoms of combat-related PTSD [SMD = −0.36, 95%CI (−0.62,−0.09)], depression [SMD = −0.28, 95%CI (−0.45,−0.10)], anxiety [SMD = −0.44, 95%CI (−0.64,−0.23)], re-experience [SMD = −0.33, 95%CI (−0.52,−0.13)], avoidance [SMD = −0.24, 95%CI (−0.43,−0.05)], and hyper-arousal [SMD = −0.26, 95%CI (−0.48,−0.03)]. Compared with the placebo, in terms of acceptability, overall active comparators did not significantly decrease all-cause discontinuance rates [RR = 0.97, 95%CI (0.78,1.20)], and the significance decreased due to AEs [RR = 2.42, 95%CI (1.41,4.13)]. Nevertheless, overall there was no statistically significant difference for overall AEs, including somnolence, sedation, dizziness, paresthesia, anxiety, blurred vision, generalized anxiety disorder, and sleep disturbance. All funnel plots were symmetrical and no publication bias was found.

**Conclusion:** Active drugs, especially amitriptyline, imipramine, and quetiapine, had a positive effect on the improvement of combat-related PTSD symptoms. Despite there being no significant increase in the AEs of the active drugs, the fact that the discontinuation rates of these drugs, including risperidone, imipramine, and topiramate, were increased deserves attention. Furthermore, as active drugs were effective across ethnic groups and battlefields, active drug regimens were revealed to be more appropriate for treating people with symptoms of extreme severe PTSD (≥80) or PTSD that is at least 8 weeks old. In addition, current evidence was from adults under 60 years of age and male combat-related PTSD. Whether this evidence can be extended to other populations of combat-related PTSD needs to be confirmed by subsequent high-quality, large-sample studies.

## 1 Introduction

Post-traumatic stress disorder (PTSD) is a kind of stress disorder with severe clinical symptoms, poor prognosis, and possible brain damage in traumatic and stress-related disorders (Mithoefer et al., 2018). PTSD refers to individuals who have faced unusually strong mental stress, such as natural disasters, traffic accidents, the sudden loss of relatives, and other accidents, which result in the emergence of stress-related disorder. The lifetime prevalence of PTSD in adults is approximately 8% (Kessler et al., 2012; Nichter et al., 2021), between 10 and 20% of trauma survivors in some cases develop chronic PTSD. This brings considerable distress to society, families, and individuals, and causes huge economic and social burdens, and even increases the risk of suicide (Kessler et al., 1995; Kessler, 2000; Frayne et al., 2004; Krysinska and Lester, 2010; Wittchen et al., 2011).

PTSD is a common clinical mental disease, especially for some high-risk groups. In Iran, which has survived 8 years of war, about 40% of veterans with PTSD have combat-related PTSD associated with more severe symptoms and extreme symptom conditions that are harder to treat PTSD (Mithoefer et al., 2018). Current research (Mohamed and Rosenheck, 2008) indicates that medication is a common option for the treatment of combat-related PTSD. Although sertraline and paroxetine are considered important and common medications for PTSD, their actual efficacy of selective serotonin reuptake inhibitors (SSRIs) remains controversial (Bajor et al., 2011). In addition, current drug regimens for PTSD do not have satisfactory clinical outcomes, with an approximately 60% response rate among PTSD patients treated with SSRIs (Suttajit et al., 2014). Related studies (Bisson et al., 2019) have focused on the efficacy of SSRI on combat-related PTSD, investigating the anxiety and depression associated with PTSD. However, the three core symptoms, including avoidance, re-experience, and hyper-arousal, based on the DMS-III have not been researched to date (Bisson et al., 2019). Other studies have found that ketamine and other drugs can affect the pathway of specific signals in suicidal tendency treatment, which achieves the effect of rapid feelings of anti-suicide and anti-depression (De Berardis et al., 2018). However, as knowledge of neuroscience and the pathophysiology of human behavior continue to advance, suicidal behavior remains a challenge. Although the possible pathophysiological factors that may explain the complex link between PTSD and suicide have been explored in studies, there is little research on the underlying role of neurobiological factors (e.g., genetics, exogenous and endogenous stressors, epigenetic, the hypothalamic-pituitary-adrenal stress-response system, the involvement of the monoaminergic neurotransmitter systems, particularly the serotonergic ones, the lipid profile), neuro-immunological biomarkers, brain-derived neurotrophic factors, and other neuromodulators (Orsolini et al., 2020). Based on the fact that treating PTSD with conventional drugs can involve major challenges, including excessive adverse effects, limited efficacy, and lower patient compliance, this study comprehensively evaluates the efficacy, acceptability, and safety of active drugs for PTSD, and explores the influence of relevant confounding factors on PTSD to ensure the reliability and extrapolation of evidence, and provide a reference for clinicians when making treatment decisions.

## 2 Methods

### 2.1 Search Strategy

The Ovid MEDLINE, Ovid EMBASE, Cochrane Library, Scopus, ScienceDirect, and Web of Science databases were searched for randomized controlled trials (RCTs), which had investigated active pharmaceutical administration in combat-related PTSD until April 21, 2021. The detailed search strategy is displayed in Supplementary Method S1.

### 2.2 Inclusion Criteria and Exclusion Criteria

The included studies met the following criteria: 1) Adult (≥18 years old) with all combat-related PTSD according to diagnostic criteria, including DSM-III, DSM-III-R, DSM-IV, DSM-IV-TR, DSM-V or ICD-10; 2) Interventions: any dosages and treatment duration of active pharmaceutical administration, including amitriptyline, aripiprazole, dexamethasone, fluoxetine, guanfacine, imipramine, olanzapine, phenelzine, prazosin, pregabalin, quetiapine, riluzole, risperidone, rivastigmin, topiramate, vilazodone, and other active drug agents; 3) Comparisons: Placebo; 4) Outcomes: effectiveness (changes in total symptoms of combat-related PTSD, depression, anxiety, re-experiencing, avoidance and hyper-arousal), acceptability [all-cause discontinuation and discontinuation due to adverse events (AEs)], and AEs (somnolence, sedation, dizziness, paresthesia, anxiety and blurred vision, generalized anxiety disorder and sleep disturbance). The change in total symptoms of combat-related PTSD according to the clinician rating scale was defined as primary outcome, and the others were defined as secondary outcomes. 5) Study design of included studies: RCTs.

Studies with one of the following criteria were excluded: 1) subjects diagnosed with schizophrenia, schizophrenia disorder, bipolar disorder, exhibiting clinically significant suicidal, homicidal ideation or other dangers; 2) a combination of treatment options other than drug intervention; 3) duplicate data or data could not be retrieved.

### 2.3 Data Extraction and Quality Assessment

Basic information was extracted, including study, year, sample, age, gender, battlefield, race, diagnostic criteria, baseline score, dosages of drugs, duration of treatment, and interesting outcomes. We used the change values from the baseline as much as possible in all the continuous outcomes. If change values from the baseline were not mentioned, we used a comparison of final measurements according to the Cochrane handbook (Julian and Sally, 2011), which is a randomized trial estimating the same baseline value in theory.

The quality assessment was employed by the risk of bias based on the Cochrane Handbook for Systematic Reviews of Interventions (Julian and Sally, 2011).

### 2.4 Statistical Analysis

Risk ratios (RR) or standardized mean difference (SMD) with 95% were employed for dichotomous data and continuous data. The heterogeneity of the meta-analyses was measured by calculating the I^2^ (Higgins et al., 2003). If I^2^ ≥ 40%, the random effects models were employed; otherwise, the fixed model was adopted. Subgroup analyses were employed for investigating the clinical benefits of overall active comparators in patients of age (<60 years and ≥60 years), race (white and other), gender (male and female), different battlefield, duration of treatment, and severity of trauma, based on CAPS scores (60–79 and ≥80) based on changes in total symptoms of combat-related PTSD, depression, and anxiety. Funnel plots were used to detect publication bias (Moher et al., 2009). All statistical analyses were employed for the software of RevMan 5.4.1. The Preferred Reporting Items of Systematic Review and Meta-Analyses (PRISMA) by systematic review and meta-analysis (Moher et al., 2009) were employed for the systematic evaluation and meta-analysis.

## 3 Results

### 3.1 Study Selection

**FIGURE 1:**
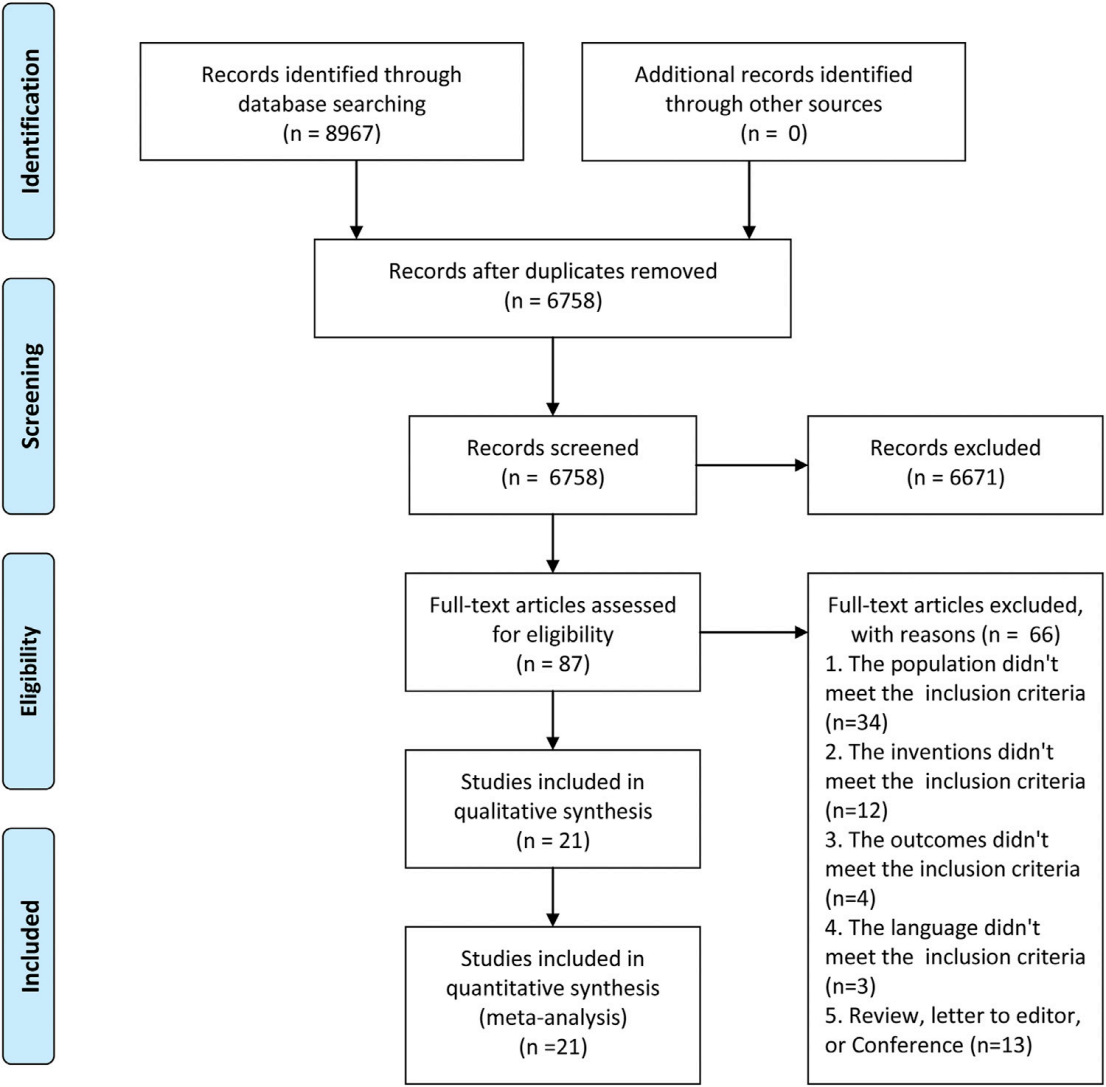
Literature screening.

As shown in the study selection in Figure 1, 8,967 studies were searched from databases, there were 6,758 studies left after deleting duplicate studies, among which this study used titles and abstracts to filter. We excluded 6,671 studies due to them having unrelated themes. A full-text review was done for the remaining 87 studies and we identified 21 articles (Davidson et al., 1990; Kosten et al., 1991; Hertzberg et al., 2000; Stein et al., 2002; Hamner et al., 2003; Monnelly et al., 2003; Akuchekian and Amanat, 2004; Bartzokis et al., 2005; Neylan et al., 2006; Lindley et al., 2007; Raskind et al., 2007; Davis et al., 2008; Baniasadi et al., 2014; Naylor et al., 2015; Petrakis et al., 2016; Villarreal et al., 2016; Ramaswamy et al., 2017; Rezaei Ardani et al., 2017; Surís et al., 2017; Raskind et al., 2018; Spangler et al., 2020) with 22 RCTs that meet the inclusion criteria for this meta-analysis.

### 3.2 Study Characteristic and Quality Assessment

In the meta-analysis, 22 RCTs with 1,221 patients provided an evaluation of the clinical benefit of drugs in combat-related PTSD. However, there were 12 RCTs in an all-male population (Kosten et al., 1991; Hertzberg et al., 2000; Stein et al., 2002; Monnelly et al., 2003; Bartzokis et al., 2005; Lindley et al., 2007; Baniasadi et al., 2014; Rezaei Ardani et al., 2017; Surís et al., 2017), and the rest were in a mixed population, lacking RCT of drug therapy in female patients. The majority of battlefields were Afghanistan or Iraq (Spangler et al., 2020), Iran or Iraq (Akuchekian and Amanat, 2004; Baniasadi et al., 2014; Rezaei Ardani et al., 2017), Vietnam (Kosten et al., 1991; Hertzberg et al., 2000; Hamner et al., 2003; Lindley et al., 2007), and unreported or different combat locations (Davidson et al., 1990; Stein et al., 2002; Bartzokis et al., 2005; Neylan et al., 2006; Raskind et al., 2007; Davis et al., 2008; Naylor et al., 2015; Petrakis et al., 2016; Villarreal et al., 2016; Ramaswamy et al., 2017; Surís et al., 2017; Raskind et al., 2018). Sample sizes for all included studies ranged from 12 to 304. A total of 16 drugs were included in this study for the treatment of combat-related chronic PTSD, among which three studies reported risperidone and three studies reported prazosin. Guanfacine and topiramat were discussed in two studies, and the other drugs, including pregabalin, amitriptyline, fluoxetine, phenelzine, aripiprazole, vilazodone, rivastigmine, riluzole, olanzapine, dexamethasone, and quetiapine, had only one study reporting work on PTSD. The other main features are displayed in Table 1. The quality assessment is presented in Supplementary Table S1.

**TABLE 1:** Summary of included clinical trials and patient characteristics.

Study, year	Sample (I/C)	Age (SD)	Gender (male, %)	Battlefield	Race (white)	Diagnostic criteria	Baseline score 773	Dosages of drugs (mg/d)	During of treatment (week)
Spangler et al. (2020)	36/38	37.8 (8.5)	63 (85.1)	Afghanistan or Iraq	47	DSM-IV	CAPS: 70.6 (19.8)/62.4(16.9)	Riluzole 100–200 mg/d	8
Akuchekian and Amanat, (2004)	34/33	39.8 (4.19)	67 (100)	Iran or Iraq	NR	DSM-IV	CAPS: 49.81 (8.42)	Topiramate 50–500 mg/d	12
Baniasadi et al. (2014)	18/19	48.16 (3.55)	37 (100)	Iran or Iraq	NR	DSM-IV-TR	PCL-M: 55.94 (7.65)	Pregabalin 75–300 mg/d	6
Bartzokis et al. (2005)	33/32	51.6 (4.2)	65 (100)	Vietnam (63), Persian Gulf (2)	44	DSM-IV	CAPS: 100.43 (13.96)	Risperidone 3 mg/d	16
Davidson et al. (1990)	25/21	49.22 (11.94)	NR	NR	NR	DSM-III	IES: 34.54 (7.59)	Amitriptyline 160.7 (50–300 mg/d)	8
Davis et al. (2008)	18/17	53.46 (7.46)	32 (91.43)	NR	25	DSM-IV	CAPS: 85.14 (17.38)	Guanfacine 1–2 mg/d	8
Hamner et al. (2003)	19/18	52.21 (6.44)	NR	Vietnam	17	DSM-IV	CAPS: 89.72 (18.31)	Risperidone 1–6 mg/d	5
Hertzberg et al. (2000)	6/6	46 (44–48)	12 (100)	Vietnam	5	DSM-IV	DTS: 108.5 (20.09)	Fluoxetine 48 (10–60 mg/d)	12
Kosten et al. (1991)	19/18	38.51 (2.04)	37 (100)	Vietnam	31	DSM III	IES: 31.77 (14.21)	Imipramine 225 (50–300 mg/d)	8
Kosten et al. (1991)	23/18	38.56 (2.04)	41 (100)	Vietnam	34	DSM III	IES 34.96 (15.26)	Phenelzine 68 mg (15–75 mg/d)	8
Lindley et al. (2007)	20/20	53.4 (0.76)	40 (100)	Vietnam	25	NR	CAPS: 61.55 (17.93)	Topiramate 50–200 mg/d	7
Monnelly et al. (2003)	7/8	51.35 (6.3)	15 (100)	Vietnam (2), Gulf war (12)	NR	DSM-IV	PCL-M: 72.47	Risperidone 0.5–2 mg/d	6
Naylor et al. (2015)	7/7	33.82 (4.81)	9 (64.29)	NR	7	DSM-IV	CAPS: 86.45 (15.37)	Aripiprazole 5–20 mg/d	10
Neylan et al. (2006)	29/34	NR	NR	NR	NR	DSM-IV	CAPS: 68.34 (20.57)	Guanfacine 0.5–3 mg/d	8
Petrakis et al. (2016)	50/46	43.97 (13.02)	89 (92.71)	NR	78	DSM-IV	CAPS: 73.78 (17.77)	Prazosin 14.5 (2–16 mg/d)	13
Ramaswamy et al. (2017)	29/30	32.7 (7.1)	57 (97)	NR	32	DSM-IV	CAPS: 75.45 (12.98)	Vilazodone 10–40 mg/d	12
Raskind et al. (2007)	20/20	26 (9)	2 (5)	Vietnam War: 32, World War II: 2, Korean War: 3, Panama invasion: 1, First Gulf War: 1	26	DSM-IV	CAPS: 77 (19.87)	Prazosin 13 (2–15 mg/d)	8
Raskind et al. (2018)	152/152	51.85 (13.78)	297 (97.7)	NR	203	DSM-IV	CAPS: 81.3 (16.3)	Prazosin (1–20 mg/d for men or 1–12 mg/d for women)	10
Rezaei Ardani et al. (2017)	12/12	50.22 (5.66)	24 (100)	Iran or Iraq	NR	DSM-IV-TR	PCL-M: 49.5 (5.85)	Rivastigmin 3–6 mg/d	12
Stein et al. (2002)	10/9	53.26 (7.44)	19 (100)	Vietnam (16), NR (3)	NR	DSM-IV	CAPS: 85.11 (19.03)	Olanzapine 15 (10–20 mg/d)	8
Surís et al. (2017)	26/28	37.5 (14.15)	54 (100)	NR	36	DSM-IV-TR	PCL: 55.85 (10.88)	Dexamethasone 0.15 mg/kg	2
Villarreal et al. (2016)	23/19	52.95 (11.07)	75 (93.75)	NR	42	DSM-IV	CAPS: 73.12 (14.24)	Quetiapine 25–800 mg/d	12

**Note:** C: placebo; CAPS: clinician-administered PTSD, scale; DSM-IV-TR: the Diagnostic and Statistical Manual of Mental Disorders, ED, 4, Text Rev; DTS: davidson trauma scale; I: active drug agent; IES-R: the Impact of Event Scale-Revised; M.I.N.I.: the Mini-International Neuropsychiatric Interview; NR: not reported; PTSD: Post-traumatic stress disorder; PCL-M: the Patient Checklist for PTSD-Military Version; SD: standard deviation; SI-PTSD: Structured Interview for Post-Traumatic Stress Disorder; SPRINT: short post traumatic stress disorder rating interview.

### 3.3 Results of Meta-Analysis

#### 3.3.1 Effectiveness

##### 3.3.1.1 Changes in Total Symptoms of Combat-Related PTSD

**FIGURE 2:**
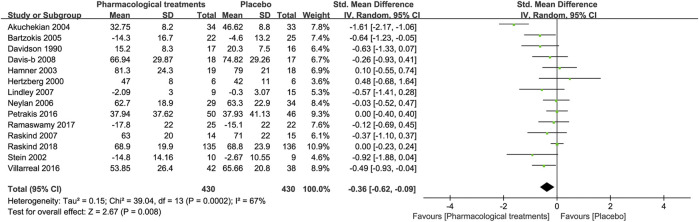
Forest for change in total symptoms of combat-related PTSD based on a clinician-assessed scale based on CAPS scores.

In terms of changes in total combat-related PTSD symptoms, seven drugs in 14 RCTs were compared with the placebo in this study. Overall active comparators [SMD = −0.36, 95%CI (−0.62, −0.09)] are shown in Figure 2 and quetiapine (SMD = −0.49, 95%CI (−0.93, −0.04)) in Table 2, which showed statistical differences. However, other active drugs, including topiramate, guanfacine, prazosin, vilazodone, risperidone, and olanzapine did not significantly reduce the change in total symptoms of combat-related PTSD, as outlined in Table 2.

**TABLE 2:** Meta-analysis of different drug classifications for efficacy outcomes.

Drugs	N	Sample (T/C)	SMD, 95%CI	Drugs	N	Sample (T/C)	SMD, 95%CI
Change in total symptoms of combat-related PTSD				Depression			
Amitriptyline	0	NR	NR	Amitriptyline	1	17/16	−1.16 (−1.90, −0.41)
Dexamethasone	0	NR	NR	Dexamethasone	1	26/28	−0.14 (−0.67, 0.40)
Guanfacine	2	47/51	−0.11 (−0.51, 0.29)	Guanfacine	1	18/17	−0.32 (−0.98, 0.35)
Olanzapine	1	10/9	−0.92 (−1.88, 0.04)	Olanzapine	0	NR	NR
Prazosin	3	199/197	−0.02 (−0.22, 0.17)	Prazosin	1	14/15	−0.26 (−0.99, 0.47)
Pregabalin	0	NR	NR	Pregabalin	1	18/19	0.22 (−0.43, 0.86)
Quetiapine	1	42/38	−0.49 (−0.93, −0.04)	Quetiapine	1	42/38	−0.63 (−1.08, −0.18)
Riluzole	0	NR	NR	Riluzole	1	36/38	0.03 (−0.42, 0.49)
Risperidone	2	41/43	−0.30 (−0.74, 0.13)	Risperidone	1	18/23	−0.27 (−0.89, 0.35)
Topiramate	1	9/15	−0.57 (−1.41, 0.28)	Topiramate	1	9/15	0.32 (−0.51, 1.16)
Vilazodone	1	25/22	−0.12 (−0.69, 0.45)	Vilazodone	1	23/24	0.06 (−0.51, 0.63)
**Anxiety**				**Re-experence**			
Amitriptyline	1	17/16	−0.99 (−1.72, −0.26)	Amitriptyline	1	17/16	−0.75 (−1.46, −0.04)
Guanfacine	0	NR	NR	Guanfacine	1	18/17	−0.10 (−0.77, 0.56)
Imipramine	1	19/18	−0.67 (−1.34, −0.01)	Imipramine	1	19/18	−1.07 (−1.77, −0.38)
Phenelzine	1	23/18	−0.46 (−1.08, 0.17)	Phenelzine	1	23/18	−0.19 (−0.80, 0.43)
Prazosin	1	18/19	−0.26 (−0.90, 0.39)	Prazosin	1	50/46	0.05 (−0.35, 0.45)
Quetiapine	1	12/19	−0.41 (−0.86, 0.03)	Quetiapine	1	42/38	−0.53 (−0.98, −0.09)
Riluzole	1	36/38	−0.16 (−0.62, 0.30)	Riluzole	0	NR	NR
Risperidone	0	NR	NR	Risperidone	2	41/43	−0.33 (−0.76, 0.11)
Rivastigmin	1	18/23	−0.82 (−1.46, −0.17)	Rivastigmin	1	12/12	−0.18 (−0.98, 0.62)
Vilazodone	1	22/24	−0.21 (−0.79, 0.37)	Vilazodone	1	15/15	0.03 (−0.69, 0.74)
**Avoidance**				**Hyper-arousal**			
Amitriptyline	1	17/16	−0.90 (−1.62, −0.18)	Amitriptyline	0	NR	NR
Guanfacine	1	18/17	−0.38 (−1.05, 0.29)	Guanfacine	1	18/17	−0.23 (−0.89, 0.44)
Imipramine	1	19/18	−0.81 (−1.49, −0.14)	Imipramine	0	NR	NR
Phenelzine	1	23/18	−0.25 (−0.87, 0.37)	Phenelzine	0	NR	NR
Prazosin	1	50/46	0.09 (−0.31, 0.49)	Prazosin	1	50/46	0.06 (−0.34, 0.46)
Quetiapine	1	42/38	−0.24 (−0.68, 0.20)	Quetiapine	1	42/38	-0.55 (-1.00, -0.10)
Risperidone	2	41/43	−0.03 (−0.46, 0.40)	Risperidone	2	41/43	−0.34 (−0.78, 0.10)
Rivastigmin	1	12/12	−0.41 (−1.22, 0.40)	Rivastigmin	1	12/12	-0.33 (-1.13, 0.48)
Vilazodone	1	15/15	−0.02 (−0.74, 0.70)	Vilazodone	1	15/15	−0.13 (−0.84, 0.59)

**Note:** NR, not reported; PTSD, Post-traumatic stress disorder; I, intervention; C, placebo; CI, confidence interval; N, number of included studies; SMD, standard mean difference.

##### 3.3.1.2 Symptom of Depression

**FIGURE 3:**
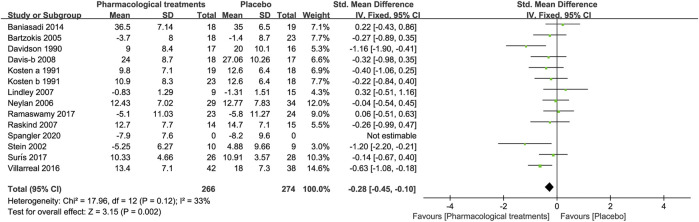
Forest for symptoms of depression.

In terms of changes in depressive symptoms, nine drugs in 14 RCTs were compared with the placebo in this study. As shown in Figure 3, overall, the active comparators [SMD = −0.28, 95%CI (−0.45, −0.10)], both amitriptyline [SMD = −1.16, 95%CI (−1.90, −0.41)] and quetiapine [SMD = −0.63, 95%CI (−1.08, −0.18)] significantly effected improved depressive symptoms, as shown in Table 2. However, other active drugs, including riluzole, topiramate, pregabalin, risperidone, guanfacine, prazosin, and dexamethasone had no statistically significant difference, as shown in Table 2.

##### 3.3.1.3 Symptom of Anxiety

**FIGURE 4:**
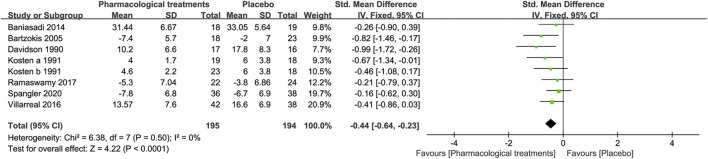
Forest for symptoms of anxiety.

In terms of changes in anxiety symptoms, seven drugs in 8 RCTs were compared with placebo in this study. Overall active comparators [SMD = −0.44, 95%CI (−0.64, −0.23)] and different drugs classifications in Figure 4, such as risperidone [SMD = −0.82, 95%CI (−1.46, −0.17)], amitriptyline [SMD = −0.99, 95%CI (−1.72, −0.26)], and imipramine [SMD = −0.67, 95%CI (−1.34, −0.01)] significantly reduced the symptoms of anxiety. However, other active drugs, including phenelzine, quetiapine, riluzole, and pregabalin had no statistically significant difference, as shown in Table 2.

##### 3.3.1.4 Symptoms of Re-Experiencing

**FIGURE 5:**
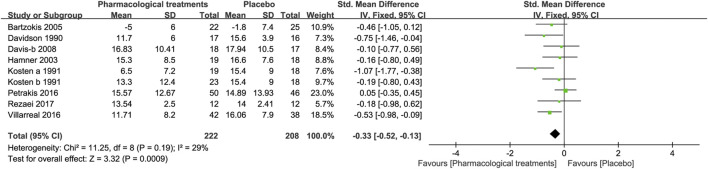
Forest for symptoms of re-experiencing.

In terms of changes in the symptoms of anxiety, eight drugs in 9 RCTs were compared with placebo in this study. As shown in Figure 5, overall, active comparators [SMD = -−0.33, 95%CI (−0.52, −0.13)], amitriptyline [SMD = −0.75, 95%CI (−1.46, −0.04)], imipramine [SMD = −1.07, 95%CI (−1.77, −0.38)], and quetiapine [SMD = −0.53, 95%CI (−0.98, −0.09)] had significance and effectively improved re-experiences of symptoms (Table 2). However, other active drugs, including risperidone, guanfacine, phenelzine, prazosin, and rivastigmine had no statistically significant difference, as indicated by Table 2.

##### 3.3.1.5 Symptoms of Avoidance

**FIGURE 6:**
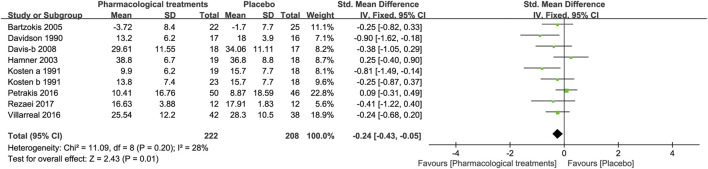
Forest for symptoms of avoidance.

Of the eight drugs in the 9 RCTs compared with placebo in this study, overall the active comparators [SMD = −0.24, 95%CI (−0.43, −0.05)] shown in Figure 6 and different drug classifications, including amitriptyline [SMD = −0.90, 95%CI (−1.62, −0.18)] and imipramine [SMD = −0.81, 95%CI (−1.49, −0.14)], significantly reduced symptoms of avoidance (see Table 2). Other active drugs, including risperidone, guanfacine, phenelzine, prazosin, rivastigmine, and quetiapine showed no statistically significant difference (see Table 2).

##### 3.3.1.6 Symptoms of Hyper-Arousal

**FIGURE 7:**
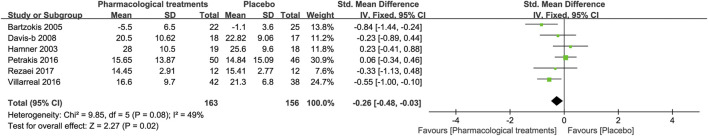
Forest for symptoms of hyper-arousal.

In terms of changes in the symptoms of anxiety, five drugs in 6 RCTs were compared with placebo. The active comparators [SMD = −0.26, 95%CI (−0.48, −0.03)] outlined in Figure 7, including quetiapine [SMD = −0.55, 95%CI (−1.00, −0.10)] are shown in Table 2. Other active drugs, including risperidone, guanfacine, prazosin, and rivastigmine, had no statistically significant difference (see Table 2).

#### 3.3.2 Acceptability

##### 3.3.2.1 All-Cause Discontinuation

**FIGURE 8:**
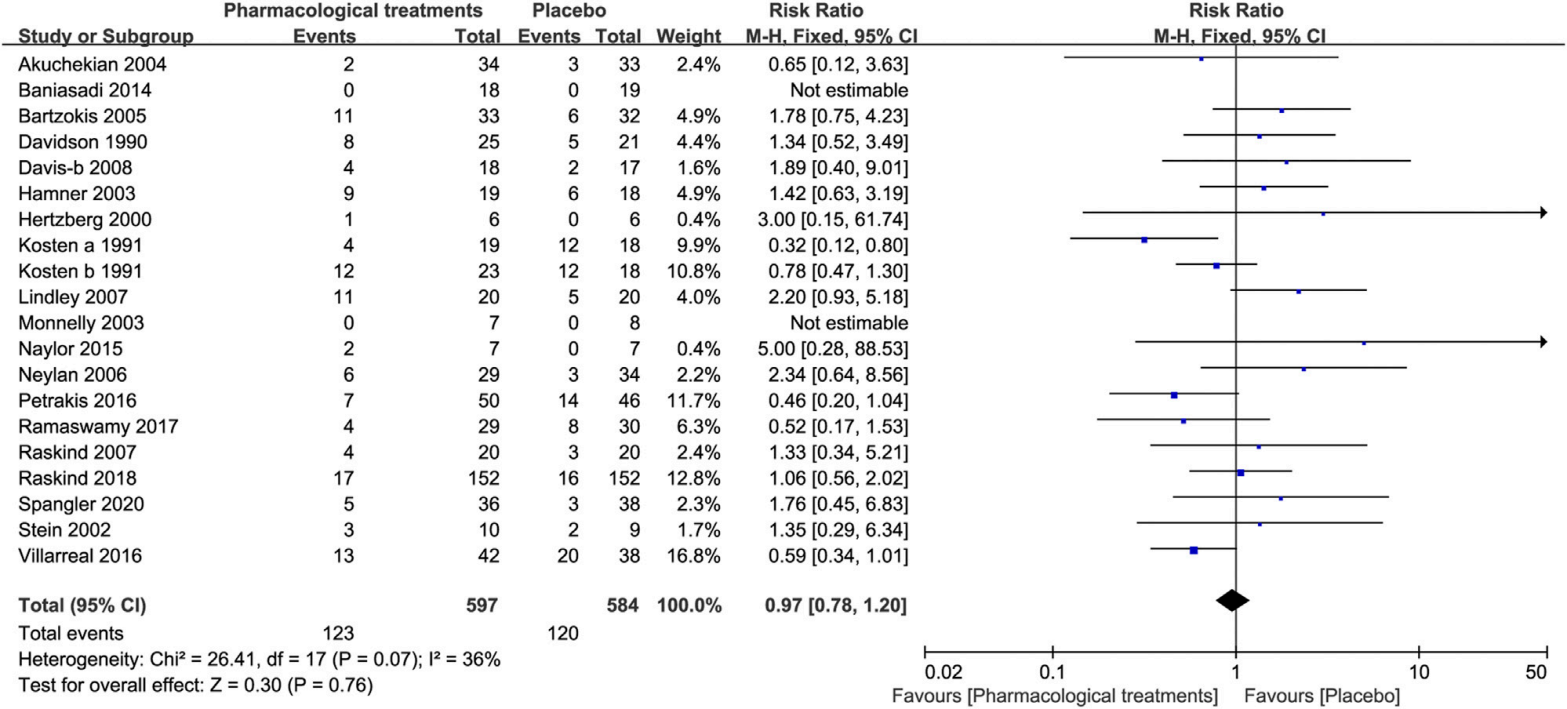
Forest for all-cause discontinuation rate.

The active comparators [RR = 0.97, 95%CI (0.78, 1.20)] shown in Figure 8, except for risperidone [RR = 1.77, 95%CI (1.09, 2.89)] and imipramine [RR = 0.32, 95%CI (0.12, 0.80)] did not show a statistically significant difference for all-cause discontinuance, as indicated by Table 3.

**TABLE 3:** Meta-analysis of different drug classifications for acceptability.

Drugs	N	Sample (T/C)	RR, 95%CI	Drugs	N	Sample (T/C)	RR, 95%CI
All-cause discontinuation rate				Discontinuation rate due to AEs			
Amitriptyline	1	25/21	1.34 (0.52, 3.49)	Amitriptyline	1	25/21	5.92 (0.32, 108.54)
Aripiprazole	1	7/7	5.00 (0.28, 88.53)	Aripiprazole	1	7/7	Not estimable
Dexamethasone	0	NR	NR	Dexamethasone	1	42/38	2.71 (0.79, 9.29)
Fluoxetine	1	6/6	3.00 (0.15, 61.74)	Fluoxetine	1	6/6	3.00 (0.15, 61.74)
Guanfacine	2	47/51	2.15 (0.79, 5.82)	Guanfacine	1	29/34	8.17 (0.44, 151.84)
Imipramine	1	19/18	0.32 (0.12, 0.80)	Imipramine	1	19/18	0.32 (0.04, 2.76)
Olanzapine	1	10/9	1.35 (0.29, 6.34)	Olanzapine	1	10/9	4.55 (0.25, 83.70)
Phenelzine	1	23/18	0.78 (0.47, 1.30)	Phenelzine	1	23/18	1.04 (0.27, 4.08)
Prazosin	3	222/218	0.83 (0.52, 1.31)	Prazosin	1	50/46	Not estimable
Pregabalin	1	18/19	Not estimable	Pregabalin	1	18/19	Not estimable
Quetiapine	1	40/38	0.59 (0.34, 1.01)	Quetiapine	0	NR	NR
Riluzole	1	36/38	1.76 (0.45, 6.83)	Riluzole	1	36/38	5.27 (0.26, 106.16)
Risperidone	3	72/70	1.77 (1.09, 2.89)	Risperidone	3	59/58	1.45 (0.26, 8.14)
Rivastigmin	0	NR	NR	Rivastigmin	1	12/12	Not estimable
Topiramate	2	57/51	0.76 (0.45, 1.27)	Topiramate	2	54/53	4.17 (1.15, 15.13)
Vilazodone	1	29/30	0.52 (0.17, 1.53)	Vilazodone	1	29/30	3.10 (0.13, 73.14)

**Note:** NR, not reported; I, intervention; C, placebo; CI, confidence interval; N, number of included studies; RR, relative risk.

##### 3.3.2.2 Discontinuation due to AEs

**FIGURE 9:**
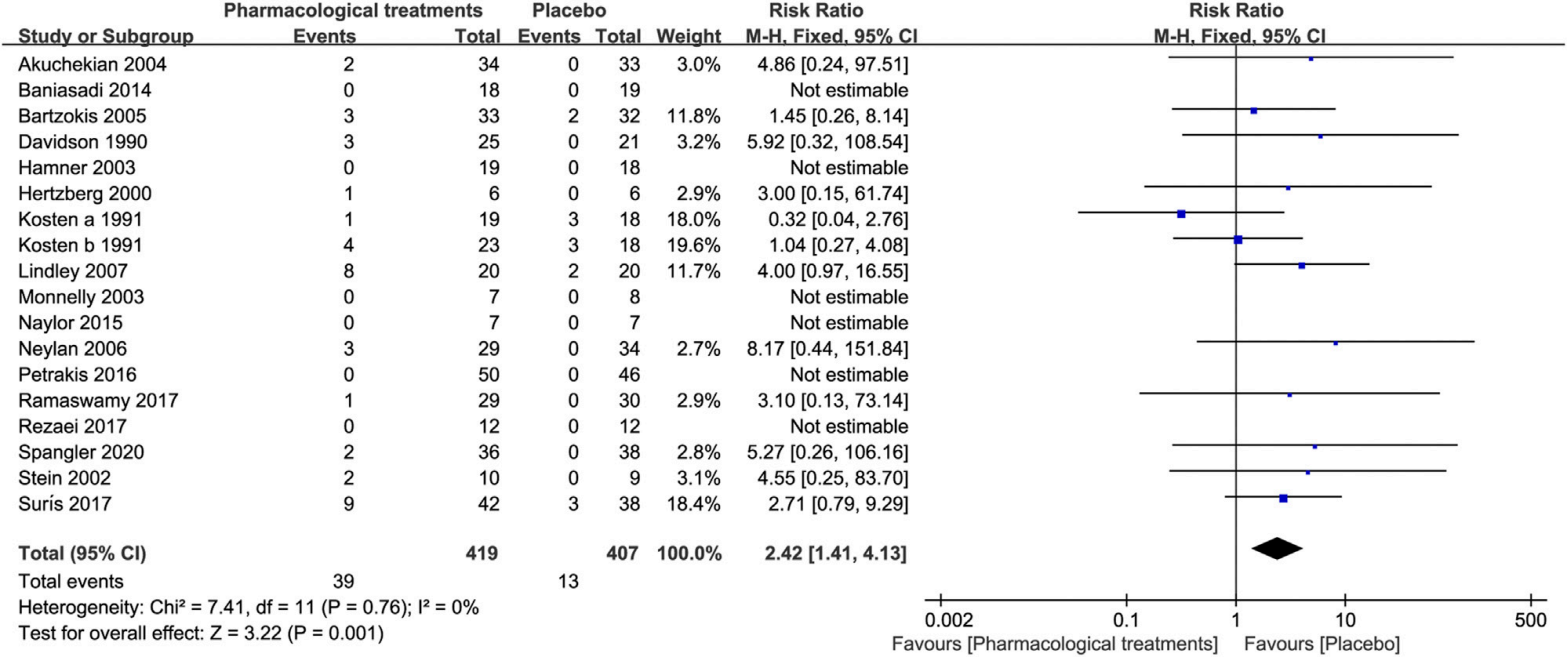
Forest for discontinuation rate due to adverse events.

The difference in the overall active comparators [RR = 2.42, 95%CI (1.41, 4.13)] discontinued due to AEs was statistically significant compared with placebo, see Figure 9. As Table 3 reveals, of the different drug classifications discontinued due to AEs, topiramate [RR = 4.17, 95%CI (1.15, 15.13)] was significantly higher than placebo, but the other active comparators were not.

#### 3.3.3 Adverse Events

Compared with placebo, overall, there was no statistically significant difference among active comparators in all AEs, including somnolence [RR = 4.55, 95%CI (0.25, 8.70)], sedation [RR = 5.00, 95%CI (0.26, 98.00)], dizziness [RR = 1.98, 95%CI (0.91, 4.31)], paresthesia [RR = 3.00, 95%CI (0.31, 69.52)], anxiety and blurred vision [RR = 0.33, 95%CI (0.01, 7.72)], generalized anxiety disorder [RR = 0.98, 95%CI (0.47, 2.04)], and sleep disturbance [RR = 1.50, 95%CI (0.28, 8.04)]) in Table 4.

**TABLE 4:** Meta-analysis for the outcome of adverse events.

Adverse events	N	Sample (I/C)	RR, 95%CI
Anxiety and blurred vision	1	20/20	0.33 (0.01,7.72)
Dizziness	3	72/70	1.98 (0.91,4.31)
Generalized anxiety disorder	2	32/29	0.98 (0.47,2.04)
Paresthesia	1	20/20	3.00 (0.31,69.52)
Sedation	1	20/20	5.00 (0.26,98.00)
Sleep disturbance	1	20/20	1.50 (0.28,8.04)
Somnolence	1	10/9	4.55 (0.25,8.70)

**Note:** RR, relative risk; I, intervention; C, placebo; CI, confidence interval, N, number of included studies.

#### 3.3.4 Stratified Analyses

##### 3.3.4.1 Different Ages

A pooling analysis of studies involving the effect of drugs on combat-related PTSD of different ages (18–60 and more than 60) showed that overall active comparators [SMD = −0.36, 95%CI (−0.62, −0.09)− can lower the changes in combat-related PTSD symptoms, depressive [SMD = −0.28, 95%CI (−0.45, −0.10)] and anxiety [SMD = −0.44, 95%CI (−0.64, −0.23)], as shown in Table 5. However, there were no patients over the age of 60 years in the RCTs.

**TABLE 5:** Stratified analyses for the change in total symptoms of combat-related PTSD, depression, and anxiety.

Stratified analyses	Change in total symptoms of combat-related PTSD			Depression			Anxiety		
	N	Sample (T/C)	SMD, 95%CI	N	Sample (T/C)	SMD, 95%CI	N	Sample (T/C)	SMD, 95%CI
Different age									
18–60	14	430/430	−0.36 (−0.62, −0.09)	14	266/274	−0.28 (−0.45, −0.10)	8	195/194	−0.44 (−0.64, −0.23)
>60	0	0	NR	0	0	NR	0	0	NR
Different age									
White	1	42/38	−0.49 (−0.93, −0.04)	1	42/38	−0.63 (−1.08, −0.18)	1	42/38	−0.41 (−0.86, 0.03)
Other	13	388/392	−0.34 (−0.63, −0.06)	13	224/236	−0.21 (−0.40, −0.03)	13	153/156	−0.44 (−0.67, −0.22)
Different gender									
Male	5	81/88	−0.74 (−1.37, −0.12)	7	123/130	−0.18 (−0.44, 0.07)	4	78/88	−0.55 (−0.87, −0.23)
Female	0	NR	NR	0	NR	NR	0	NR	NR
Different battlefield									
Afghanistan or Iraq war	0	NR	NR	1	36/38	0.03 (−0.42, 0.49)	1	36/38	−0.16 (−0.62, 0.30)
Iran or Iraq war	1	34/33	−1.61 (−2.17, −1.06)	1	18/19	0.22 (−0.43, 0.86)	1	18/19	−0.26 (−0.90, 0.39)
Vietnam war	3	34/39	−0.04 (−0.58, 0.49)	3	51/51	−0.17 (−0.56, 0.23)	2	42/36	−0.56 (−1.01, −0.10)
Other	11	368/364	−0.17 (−0.31, −0.02)	9	197/204	−0.32 (−0.53, −0.10)	4	99/101	−0.55 (−0.87, −0.22)
Duration of Treatment									
≤8 weeks	5	92/100	−0.21 (−0.50, 0.08)	11	219/227	−0.23 (−0.46, 0.01)	5	113/109	−0.44 (−0.72, −0.15)
>8 weeks	9	338/330	−0.27 (−0.42, −0.11)	3	83/85	−0.31 (−0.73, 0.11)	3	82/85	−0.45 (−0.76, −0.14)
Severity of Trauma Based on CAPS Scores									
Severe PTSD symptoms (60–79)	6	160/170	−0.20 (−0.42, 0.01)	6	135/164	−0.13 (−0.40, 0.14)	3	100/100	−0.27 (−0.55, 0.01)
Extreme severe PTSD symptoms (≥80)	5	204/205	−0.12 (−0.31, 0.08)	4	60/64	−0.40 (−0.76, −0.04)	1	18/23	−0.82 (−1.46, −0.17)

**Note:** NR, not reported; I, intervention; C, placebo; CI, confidence interval; N, number of included studies; SMD, standard mean difference.

##### 3.3.4.2 Different Races

Compared with placebo, overall active comparators did significantly reduce the change in total symptoms of combat-related PTSD in study participants of white [SMD = −0.49, 95%CI (−0.93, −0.04)] and other races [SMD = −0.34, 95%CI (−0.63, −0.06)]. Moreover, compared with placebo for depressive symptoms, overall active comparators had significant statistical differences in participants of white [SMD = −0.63, 95%CI (−1.08, −0.18)] and other races [SMD = −0.21, 95%CI (−0.40, −0.03)]. Overall active comparators in white [SMD = −0.41, 95%CI (−0.86, 0.03)] participants had no significant relief of anxiety symptoms, but it had a significant clinical benefit for anxiety [SMD = −0.44, 95%CI (−0.67, −0.22)] in participants of other races in Table 5.

##### 3.3.4.3 Different Gender

Treatment of overall active comparators [SMD = −0.74, 95%CI (−1.37, −0.12)] significantly reduced the change in total symptoms of combat-related PTSD for male adults. Furthermore, overall active comparators [SMD = −0.18, 95%CI (−0.44, 0.07)] did not significantly relieve depressive symptoms. However, overall, active comparators [SMD = −0.55, 95%CI (−0.87, −0.23)] had significant therapeutic effects for anxiety in Table 5. Unfortunately, no RCT for female participants was involved in the systematic evaluation and meta-analysis, there was no evidence of drug treatment in female participants with combat-related PTSD.

##### 3.3.4.4 Different Battlefield

Based on various battlefields, the change in total symptoms of combat-related PTSD compared with placebo showed that overall, active comparators for various battlefields, including the Iraq or Iran war [SMD = −1.61, 95%CI (−2.17, −1.06)] and unknown battlefields [SMD = −0.17, 95%CI (−0.31, −0.02)], could significantly lower the changes in total symptoms of combat-related PTSD, but for Vietnam war battlegrounds [SMD = −0.04, 95%CI (−0.58, 0.49)] there was no relief of the change in total symptoms of combat-related PTSD. Based on various battlefields, depression compared with placebo indicated comparative battlefields which had no significant difference for depression, including Afghanistan or Iraq [SMD = 0.03, 95%CI (−0.42, 0.49)], Iraq or Iran [SMD = 0.22, 95%CI (−0.43, 0.86)], and the Vietnam war [SMD = −0.17, 95%CI (−0.56, 0.23)], but overall, active comparators for unknown battlefields [SMD = −0.32, 95%CI (−0.53, −0.10)] could relieve depression. In terms of anxiety, compared with placebo the overall active comparators for various battlefields, including Afghanistan or Iraq [SMD = −0.16, 95%CI (−0.62, 0.30)] and Iraq-Iran [SMD = −0.26, 95%CI (−0.90, 0.39)], had no significant statistical difference, but overall active comparators for the Vietnam war [SMD = −0.56, 95%CI (−1.01, −0.10)] and unknown battlefields [SMD = −0.55, 95%CI (−0.87, −0.22)] showed improved anxiety, see Table 5.

##### 3.3.4.5 Duration of Treatment

Compared with placebo, overall active comparators for a duration of treatment of less than 8 weeks [SMD = −0.21, 95%CI (−0.50, 0.08)] did not significantly lower changes to total symptoms of combat-related PTSD. However, overall active comparators [SMD = −0.27, 95%CI (−0.42, −0.11)] in the duration of drug of more than 8 weeks can significantly lower the change in total symptoms of combat-related PTSD. Furthermore, Compared with placebo for depressive, overall active comparators of less than 8 weeks [SMD = −0.23, 95%CI (−0.46, 0.01)] and more than 8 weeks [SMD = −0.31, 95%CI (−0.73, 0.11)] had no significant statistical differences. For depressive symptoms, compared with the placebo, overall active comparators of less than 8 weeks [SMD = −0.44, 95%CI (−0.72, −0.15)] and more than 8 weeks [SMD = −0.45, 95%CI (−0.76, −0.14)] duration had significant statistical differences, as seen in Table 5.

##### 3.3.4.6 Severity of Trauma

Overall active comparators in severe PTSD symptoms (60–79) [SMD = −0.20, 95%CI (−0.42, 0.01)] and extremely severe PTSD symptoms (≥80) [SMD = −0.12, 95%CI (−0.31, 0.08)] had not significantly lowered the change in total symptoms of combat-related PTSD. Moreover, compared with placebo in depression, overall active comparators in severe PTSD symptoms (60–79) [SMD = −0.13, 95%CI (−0.40, 0.14)] did not show significant improvement, but overall active comparators with extreme severe PTSD symptoms (≥80) [SMD = −0.40, 95%CI (−0.76, −0.04)] can improve depression. Compared with placebo, overall active comparators in severe PTSD symptoms (60–79) [SMD = −0.27, 95%CI (−0.55, 0.01)] did not significantly improve anxiety, but overall active comparators with extreme severe PTSD symptoms (≥80) [SMD = −0.82, 95%CI (−1.46, −0.17)] can relieve anxiety, as shown in Table 5.

### 3.4 Publication Bias

All funnel plots were symmetrical and no publication bias was found, as indicated in Supplemental Figures 1–8.

## 4 Discussion

A total of 1,263 populations of combat-related PTSD participated in 22 RCTs were included in our evaluation of the clinical benefit of drugs in treating combat-related PTSD. Studies investigating the correlation of the different underlying factors of PTSD have found that the symptomatic characteristics of anxiety and depression appeared to be more closely related to factors reflecting general anxiety than to more specific aspects of PTSD that reflect re-experience, avoidance, and hyper-arousal. This study found that guanfacine and quetiapine improved depressive symptoms positively, while risperidone, amitriptyline, and imipramine had positive effects on anxiety. Guanfacine is also an α2-adrenoreceptor agonist commonly used to treat high blood pressure and attention deficit hyperactivity disorder. However, the nightmares and sleep disturbances caused by traumatic episodes can also benefit. Because of its long half-life, it was more popular than the other adrenergic drugs mentioned above. Quetiapine’s properties as a partial agonist of 5-HT1A and as a potent inhibitor of norepinephrine transporter through its metabolite seem to partly explain the mechanism of major depressive disorder treatment (Cross et al., 2016). The receptor binding properties of quetiapine were complex and it seems impossible to explain the effects observed in major depression and anxiety disorders by a single mechanism. In addition, the amitriptyline, imipramine, and quentiapine can effectively relieve the re-experiencing symptoms. The amitriptyline and quetiapine had good clinical benefits for avoidance, and the quetiapine had a good therapeutic effect on hyper-arousal symptoms. It is important to note that quetiapine is effective against bipolar depression (Suttajit et al., 2014), and the present study speculates that this may be one of the reasons why quetiapine was effective depressive symptoms. Furthermore, TCAs, including amitriptyline and imipramine, were absorbed rapidly and distributed in large quantities, and its elimination can be delayed for 10 days (Abdollahi and Mostafalou, 2014). They acted as catecholamines reuptake inhibitors, including serotonin and norepinephrine, but they also had anticholinergic, antihistamine, and quinine effects. It is worth noting that cardiotoxicity was the main adverse effect of excessive use of TCAs, and sodium bicarbonate was used to treat poisoning. This was different from other studies (Puetz et al., 2015) that looked at medications for anxiety and depression, in which treatment with SSRIs and TCAs have been found to reduce PTSD, anxiety, and depression symptoms in veterans. SSRIs also play a role in the symptom improvement of PTSD, which was derived from the mechanism that SSRIs were neurotransmitters were released by synaptic vesicles in neurons and act on postsynaptic neurons. Neurotransmitter release was quantum release, synaptic vesicles were not released one by one, nor were they released in groups, they were released all at once and hydrolyzed rapidly. When released, it acted on the postsynaptic membrane, causing the next neuron to fire. The simplest way to do this was to reabsorb the neurotransmitters that were released, which is how neurotransmitters reabsorb. SSRI drugs acted on the reabsorption process to prevent its reuptake and continue to act on postsynaptic neurons to increase the concentration of SSRIs in the synaptic cleft, thus playing a role of anti-depression and anti-anxiety.

NICE Guidelines recommend the use of antipsychotics for people with PTSD (Excellence NIfHaC, 2019). PTSD comorbidities (McCauley et al., 2012) are treated differently for non-veterans, with higher rates of suicide attempts and poorer treatment adherence (McCauley et al., 2012; Reisman, 2016). Therefore, atypical antipsychotics may be considered more appropriate for this particular group of veterans than SSRIs. The results of the meta-analysis suggested that atypical antipsychotics improved the treatment of combat-related PTSD. This finding differs from a recent meta-analysis of the effects of drugs on PTSD. This study found that risperidone and olanzapine (Stein et al., 2002; Bartzokis et al., 2005) were suitable for veterans with extremely severe symptoms of PTSD, while there were no effective medications for veterans with severe PTSD.

In this meta-analysis, age and race were used as influencing factors for drug treatment of combat-related PTSD. In this study, drugs showed a good therapeutic effect on veterans between 18 and 60 years old, but evidence on veterans over 60 years old was lacking. The main cause appears to be women’s reported lower levels of military readiness, weaker unit cohesion, and higher incidences of depressive symptoms (Bisson et al., 2019). Unfortunately, no studies reported the treatment of female participants with drugs. In addition, the study found that anxiety can be effectively reduced in veterans when they receive medication in an unknown battlefield setting, as did those with anxiety who served in Vietnam (VA/DOD, 2018). However, overall, active comparators were not effective at treating anxiety symptoms experienced by veterans of the war in Afghanistan and Iraq, or Iran and Iraq.

Considering the efficacy of different durations of treatment, long-term treatment interventions had a more significant effect on change in total symptoms of combat-related PTSD and could reduce the clinical effect of change in total symptoms of combat-related PTSD. However, the duration of intervention showed no clinical advantage in improving depression. In contrast, interventions of different duration did not affect the overall outcome of anxiety and all reduced anxiety symptoms. In addition to cumulative therapeutic efficacy, the differences in duration of treatment may also be related to the low sample size allocation caused by stratified analysis, which reduces the power of statistical efficacy in each subgroup. Although many factors can influence the outcome to benefit analysis, the present study recommends that clinicians continue treatment for at least 8 weeks to better manage symptoms associated with PTSD.

Imipramine was the only drug that proved to be significantly superior in terms of the rate of discontinuation for all causes (Kosten et al., 1991). Imipramine is a tricyclic antidepressant, mainly used for the treatment of major depression, obsessive-compulsive disorder, generalized anxiety disorder, and other psychiatric disorders, which may be one of the reasons for the low withdrawal rate of imipramine due to AEs (Dean, 2012). After subgroup analysis, a study by Bartzokis, Hamner, and Monnelly showed a high effect on the all-cause discontinuation rate of risperidone (Hamner et al., 2003; Monnelly et al., 2003; Bartzokis et al., 2005). The high rate of risperidone withdrawal may be related to the dosage of risperidone. In related studies, risperidone was used in small doses with fewer side effects (Monnelly et al., 2003). In addition, the number of discontinuation due to AEs of anticonvulsive drugs increased significantly. This study also found that overall active comparators had certain AEs, including drowsiness, sedation, dizziness, paresthesias, anxiety and blurred vision, generalized anxiety disorder, and sleep disturbances. Lethargy, dizziness, sedation, and other AEs that occur from the use of quetiapine, and these AEs were paid attention to by clinicians.

Based on current guidelines and evidence, this study aims to make the following recommendations for clinicians when conducting research on combat-related PTSD. Clinicians should fully consider individual factors, including different symptoms, different populations groups, or severe sub-symptoms, when choosing drugs. Clinicians can also use the results of this study to initially identify appropriate drug treatment regimens, taking into account patients’ preferences (McHugh et al., 2013; Zoellner et al., 2019). For future research, the pharmacological treatment of PTSD may need to be studied with specific drugs that have shown strong therapeutic value in small sample studies. Future studies should continue to look at this particular population of combat-related PTSD and provide a rigorous stratified analysis of baseline symptoms to identify individual differences between medications.

This study has the following limitations. First of all, despite the large number of active drugs included, the sample size of included active drugs was too small, which may lead to the low statistical power of the results in this study. Therefore, the conclusions of this study need to be verified by larger samples and high-quality RCTs in the future. Secondly, there were differences in the use of certain dosages of active drugs, which causes heterogeneity in the results and influences the accuracy of the results to some extent. Finally, the SD of continuous outcomes was missing and unavailable, which was also one of the reasons that the number of studies in this analysis was too small and affected the results.

## 5 Conclusion

Active drugs, especially amitriptyline, imipramine, and quetiapine, had a positive effect and improved combat-related PTSD symptoms. Even though there was no significant increase in AEs of the active drugs, the fact that the discontinuation rates of these drugs, including risperidone, imipramine, and topiramate, were increased still deserves attention. Furthermore, active drugs were effective across ethnic groups and battlefields, and active drug regimens were more appropriate for treating people with symptoms of extreme severe PTSD (≥80) or at least 8 weeks old. In addition, the current evidence was only from male adults under 60 years of age experiencing combat-related PTSD, whether this evidence can be extended to other populations with combat-related PTSD needs to be confirmed by subsequent high-quality, large-sample studies.

## Data Availability

The original contributions presented in the study are included in the article/Supplementary Material, further inquiries can be directed to the corresponding authors.
