# Supplementary material for: Effectiveness, Acceptability and Safety of Pharmaceutical Management for Combat-Related PTSD in Adults Based on Systematic Review of Twenty-Two Randomized Controlled Trials
Source: Front Pharmacol. 2022 Jan 18;12:805354. doi: 10.3389/fphar.2021.805354 (PMC8804358; doi:10.3389/fphar.2021.805354)

**Legend of Supplementary Materials**

**Supplementary Method 1** Search strategy

**Supplemental Table 1** Risk of bias for included studies

**Supplemental Figure 1** Forest for change in total symptoms of combat-related PTSD based on clinician-assessed scale based on CAPS scores

**Supplemental Figure 2** Forest for symptoms of depression

**Supplemental Figure 3** Forest for symptoms of anxiety

**Supplemental Figure 4** Forest for symptoms of re-experiencing

**Supplemental Figure 5** Forest for symptoms of avoidance

**Supplemental Figure 6** Forest for symptoms of hyper-arousal

**Supplemental Figure 7** Forest for all-cause discontinuation rate

**Supplemental Figure 8** Forest for discontinuation rate due to AEs

**Supplementary Method 1** Search strategy

**1. Ovid MEDLINE(R) and Epub Ahead of Print, In-Process & Other Non-Indexed Citations, Daily, and Versions(R)** **<1946 to April 21, 2021>**

1. (Stress Disorders, Post-Traumatic).mp.
2. (post-traumatic stress disorder).mp.
3. (post-traumatic stress disorders).mp.
4. (disorder* AND post-traumatic).ti,ab.
5. (Stress Disorders, Traumatic).mp.
6. (Combat Disorders).mp.
7. PTSD.mp.
8. #1 OR #2 OR #3 OR #4 OR #5 OR #6 OR #7
9. Veterans.mp.
10. Veteran.mp.
11. Troops.mp.
12. War.mp.
13. Military.mp.
14. Ex-military.mp.
15. Army.mp.
16. Soldier.mp.
17. Soldiers.mp.
18. Peacemaker.mp.
19. Peacemakers.mp.
20. #9 OR #10 OR #11 OR #12 OR #13 OR #14 OR #15 OR #16 OR #17 OR #18 OR #19
21. Benzodiazepines.mp.
22. (Antidepressive Agents, Tricyclic).mp.
23. Anticonvulsants.mp.
24. (Adrenergic alpha-Antagonists).mp.
25. (Antipsychotic Agents).mp.
26. (Antidepressive Agents).mp.
27. (citalopram OR escitalopram OR fluoxetine OR fluvoxamine OR paroxetine OR sertraline OR desvenlafaxine OR venlafaxine OR duloxetine OR imipramine OR amitriptyline OR desipramine OR bupropion OR mirtazapine OR nefazodone OR trazodone OR prazosin OR olanzapine OR risperidone OR benzodiazepines OR alprazolam OR diazepam OR lorazepam OR clonazepam OR topiramate OR tiagabine OR lamotrigine OR carbamazepine OR divalproex).mp.
28. #21 OR #22 OR #23 OR #24 OR #25 OR #26 OR #27
29. #8 AND #20 AND #28
30. Limit #29 to randomized controlled trial

**2. Ovid EMbase <1946 to April 21, 2021>**

1. (Stress Disorders, Post-Traumatic).mp.
2. (post-traumatic stress disorder).mp.
3. (post-traumatic stress disorders).mp.
4. (disorder* AND post-traumatic).ti,ab.
5. (Stress Disorders, Traumatic).mp.
6. (Combat Disorders).mp.
7. PTSD.mp.
8. #1 OR #2 OR #3 OR #4 OR #5 OR #6 OR #7
9. Veterans.mp.
10. Veteran.mp.
11. Troops.mp.
12. War.mp.
13. Military.mp.
14. Ex-military.mp.
15. Army.mp.
16. Soldier.mp.
17. Soldiers.mp.
18. Peacemaker.mp.
19. Peacemakers.mp.
20. #9 OR #10 OR #11 OR #12 OR #13 OR #14 OR #15 OR #16 OR #17 OR #18 OR #19
21. Benzodiazepines.mp.
22. (Antidepressive Agents, Tricyclic).mp.
23. Anticonvulsants.mp.
24. (Adrenergic alpha-Antagonists).mp.
25. (Antipsychotic Agents).mp.
26. (Antidepressive Agents).mp.
27. (citalopram OR escitalopram OR fluoxetine OR fluvoxamine OR paroxetine OR sertraline OR desvenlafaxine OR venlafaxine OR duloxetine OR imipramine OR amitriptyline OR desipramine OR bupropion OR mirtazapine OR nefazodone OR trazodone OR prazosin OR olanzapine OR risperidone OR benzodiazepines OR alprazolam OR diazepam OR lorazepam OR clonazepam OR topiramate OR tiagabine OR lamotrigine OR carbamazepine OR divalproex).mp.
28. #21 OR #22 OR #23 OR #24 OR #25 OR #26 OR #27
29. #8 AND #20 AND #28
30. Limit #29 to randomized controlled trial

**3. CENTRAL, The** **Cochrane Library <Issue 4 of 12, April 2021>**

1. MeSH descriptor: [Stress Disorders, Post-Traumatic] explode all trees
2. (post-traumatic stress disorder):ti,ab,kw
3. (post-traumatic stress disorders):ti,ab,kw
4. (disorder* AND post-traumatic):ti,ab,kw
5. MeSH descriptor: [Stress Disorders, Traumatic] explode all trees
6. MeSH descriptor: [Combat Disorders] explode all trees
7. (PTSD):ti,ab,kw
8. #1 OR #2 OR #3 OR #4 OR #5 OR #6 OR #7
9. MeSH descriptor: [Veterans] explode all trees
10. (Veteran):ti,ab,kw
11. (Troops):ti,ab,kw
12. MeSH descriptor: [War] explode all trees
13. MeSH descriptor: [Military] explode all trees
14. (Ex-military):ti,ab,kw
15. (Army):ti,ab,kw
16. MeSH descriptor: [Military Personnel] explode all trees
17. (Soldier):ti,ab,kw
18. (Peacemaker):ti,ab,kw
19. #9 OR #10 OR #11 OR #12 OR #13 OR #14 OR #15 OR #16 OR #17 OR #18
20. MeSH descriptor: [Benzodiazepines] explode all trees
21. MeSH descriptor: [Antidepressive Agents, Tricyclic] explode all trees
22. MeSH descriptor: [Anticonvulsants] explode all trees
23. MeSH descriptor: [Adrenergic alpha-Antagonists] explode all trees
24. MeSH descriptor: [Antipsychotic Agents] explode all trees
25. MeSH descriptor: [Antidepressive Agents] explode all trees
26. MeSH descriptor: [Adrenergic alpha-Antagonists] explode all trees
27. (citalopram OR escitalopram OR fluoxetine OR fluvoxamine OR paroxetine OR sertraline OR desvenlafaxine OR venlafaxine OR duloxetine OR imipramine OR amitriptyline OR desipramine OR bupropion OR mirtazapine OR nefazodone OR trazodone OR prazosin OR olanzapine OR risperidone OR benzodiazepines OR alprazolam OR diazepam OR lorazepam OR clonazepam OR topiramate OR tiagabine OR lamotrigine OR carbamazepine OR divalproex):ti,ab,kw
28. #20 OR #21 OR #22 OR #23 OR #24 OR #25 OR #26 OR #27
29. #8 AND #19 AND #28 in Cochrane Central Register of Controlled Trials

**4. Scopus <April 21, 2021>**

TITLE-ABS-KEY ((Post Traumatic Stress Disorder) OR Posttraumatic OR (Post trauma) OR PTSD) AND (Veteran OR Troops OR War OR Military OR Ex-military OR Army OR Soldier OR Peacemaker) AND (Benzodiazepines OR (Antidepressive Agents, Tricyclic) OR Anticonvulsants OR (Adrenergic alpha-Antagonists) OR (Antipsychotic Agents) OR (Antidepressive Agents) OR citalopram OR escitalopram OR fluoxetine OR fluvoxamine OR paroxetine OR sertraline OR desvenlafaxine OR venlafaxine OR duloxetine OR imipramine OR amitriptyline OR desipramine OR bupropion OR mirtazapine OR nefazodone OR trazodone OR prazosin OR olanzapine OR risperidone OR benzodiazepines OR alprazolam OR diazepam OR lorazepam OR clonazepam OR topiramate OR tiagabine OR lamotrigine OR carbamazepine OR divalproex)

**5. ScienceDirect <April 21, 2021>**

Keywords: (Post Traumatic Stress Disorder) AND (Veteran OR Troops) AND (Benzodiazepines OR (Antidepressive Agents, Tricyclic) OR Anticonvulsants OR (Adrenergic alpha-Antagonists) OR (Antipsychotic Agents) OR (Antidepressive Agents))

**6. Web of Science (ISI) <April 21, 2021>**

1. Topic: (PTSD)

Databases= SCI-EXPANDED, SSCI, A&HCI, CPCI-S, CPCI-SSH, BKCI-S, BKCI-SSH, ESCI, CCR-EXPANDED, IC Timespan= 1970-2019

1. Topic: (posttraumatic)

Databases= SCI-EXPANDED, SSCI, A&HCI, CPCI-S, CPCI-SSH, BKCI-S, BKCI-SSH, ESCI, CCR-EXPANDED, IC Timespan= 1970-2019

1. Topic: (post trauma)

Databases= SCI-EXPANDED, SSCI, A&HCI, CPCI-S, CPCI-SSH, BKCI-S, BKCI-SSH, ESCI, CCR-EXPANDED, IC Timespan= 1970-2019

1. Topic: (Veteran)

Databases= SCI-EXPANDED, SSCI, A&HCI, CPCI-S, CPCI-SSH, BKCI-S, BKCI-SSH, ESCI, CCR-EXPANDED, IC Timespan= 1970-2019

1. Topic: (Armed Conflicts)

Databases= SCI-EXPANDED, SSCI, A&HCI, CPCI-S, CPCI-SSH, BKCI-S, BKCI-SSH, ESCI, CCR-EXPANDED, IC Timespan= 1970-2019

1. #5 OR #4 OR #3 OR #2 OR #1

Databases= SCI-EXPANDED, SSCI, A&HCI, CPCI-S, CPCI-SSH, BKCI-S, BKCI-SSH, ESCI, CCR-EXPANDED, IC Timespan= 1970-2019

1. Topic: (pharmacotherapy)

Databases= SCI-EXPANDED, SSCI, A&HCI, CPCI-S, CPCI-SSH, BKCI-S, BKCI-SSH, ESCI, CCR-EXPANDED, IC Timespan= 1970-2019

1. #6 AND #7

Databases= SCI-EXPANDED, SSCI, A&HCI, CPCI-S, CPCI-SSH, BKCI-S, BKCI-SSH, ESCI, CCR-EXPANDED, IC Timespan=1970-2019

**Supplemental Table 1** Risk of bias for included studies

| **Study, Year** | **Random sequence generation** | **Allocation concealment** | **Blinding of participants and personnel** | **Blinding of outcome assessment** | **Selective reporting** | **Incomplete outcome data** | **Other bias** |
| --- | --- | --- | --- | --- | --- | --- | --- |
| Akuchekian 2004 | Unclear | Low risk | Low risk | Unclear | Unclear | Low risk | Unclear |
| Baniasadi 2014 | Unclear | Unclear | Low risk | Unclear | Unclear | Low risk | Unclear |
| Bartzokis 2005 | Unclear | Unclear | Low risk | Low risk | Unclear | Low risk | Unclear |
| Davidson 1990 | Unclear | Unclear | Low risk | Unclear | Unclear | Low risk | Unclear |
| Davis 2008 | Unclear | Unclear | Low risk | Unclear | Unclear | Low risk | Unclear |
| Hamner 2003 | Unclear | Unclear | Low risk | Low risk | Unclear | Low risk | Unclear |
| Hertzberg 2000 | Unclear | Unclear | Low risk | Unclear | Unclear | Low risk | Unclear |
| Kosten 1991 | Unclear | Unclear | Low risk | Low risk | Unclear | Low risk | Unclear |
| Lindley 2007 | Unclear | Unclear | Low risk | Low risk | Unclear | Low risk | Unclear |
| Monnelly 2003 | Unclear | Unclear | Low risk | Low risk | Unclear | Low risk | Unclear |
| Naylor 2015 | Unclear | Unclear | Low risk | Unclear | Low risk | Low risk | Unclear |
| Neylan 2006 | Unclear | Unclear | Low risk | Low risk | Unclear | Low risk | Unclear |
| Petrakis 2016 | Unclear | Unclear | Low risk | Unclear | Low risk | Low risk | Unclear |
| Ramaswamy ‎2017 | Low risk | Unclear | Low risk | Low risk | Unclear | Low risk | Unclear |
| Raskind 2007 | Low risk | Unclear | Low risk | Low risk | Unclear | Low risk | Unclear |
| Raskind 2018 | Low risk | Low risk | Low risk | Low risk | Low risk | Low risk | Unclear |
| Rezaei 2017 | Low risk | Low risk | Low risk | Low risk | Unclear | Low risk | Unclear |
| Suris 2017 | Unclear | Unclear | Low risk | Unclear | Unclear | Low risk | Unclear |
| Villarreal 2016 | Low risk | Unclear | Low risk | Unclear | Low risk | Low risk | Unclear |
| Stein 2002 | Unclear | Unclear | Low risk | Unclear | Unclear | Unclear | Unclear |
| Spangler 2020 | Low risk | Low risk | Low risk | Low risk | Low risk | Low risk | Unclear |

**Supplemental Figure 1** Forest for change in total symptoms of combat-related PTSD based on clinician-assessed scale based on CAPS scores

**
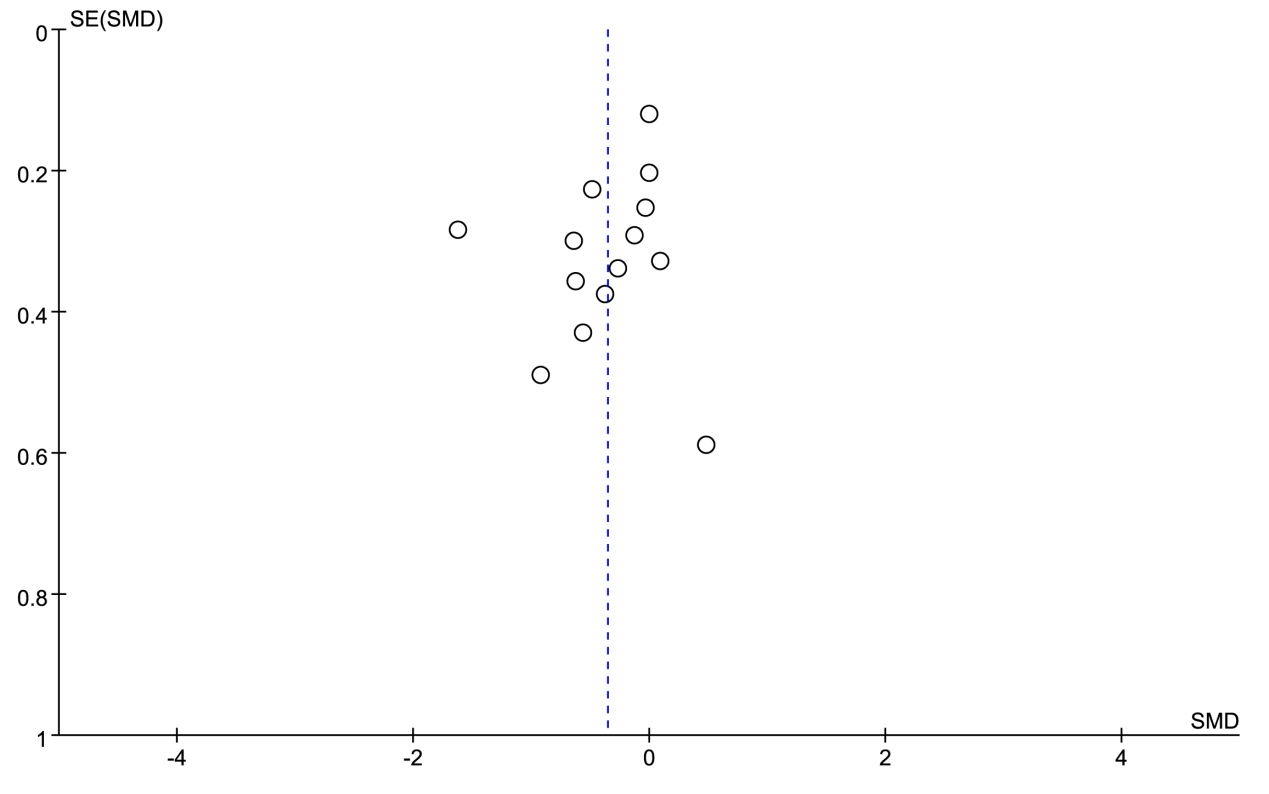
**

**Supplemental Figure 2** Forest for symptoms of depression

**
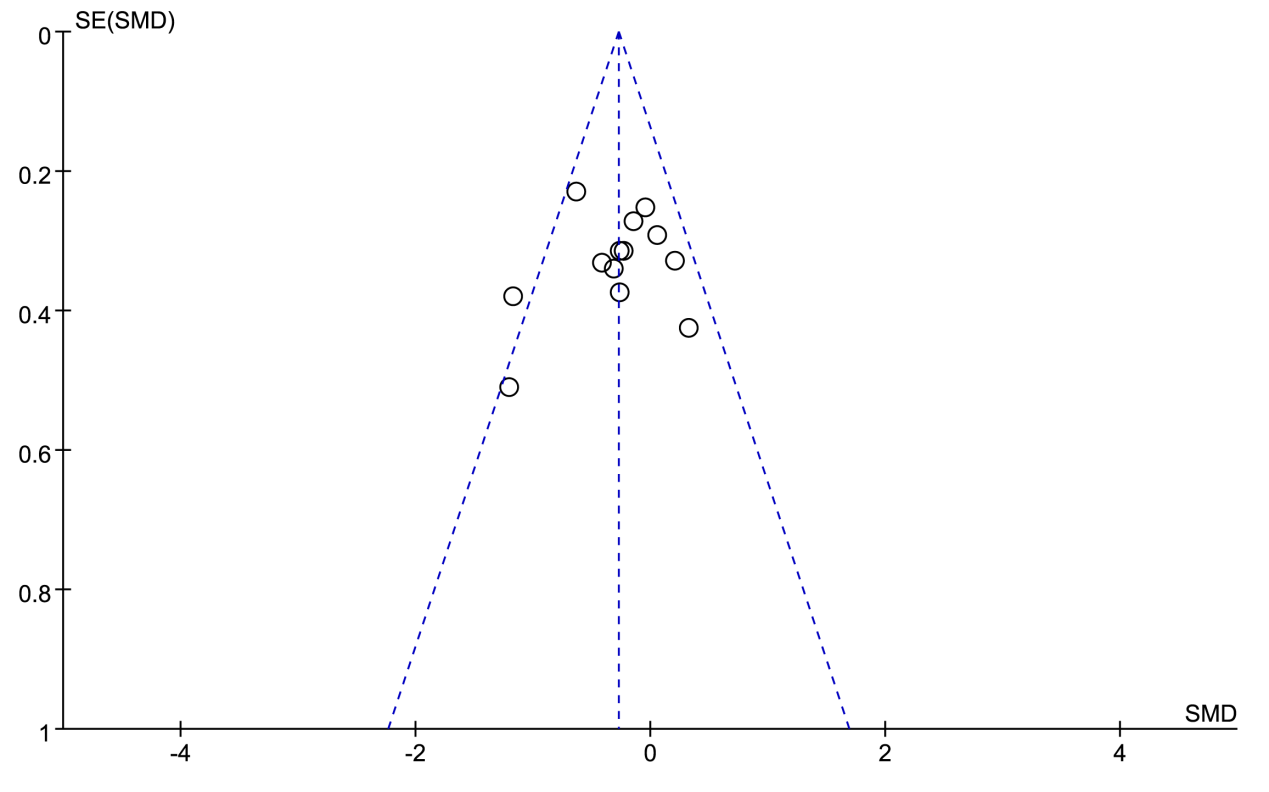
**

**Supplemental Figure 3** Forest for symptoms of anxiety

**
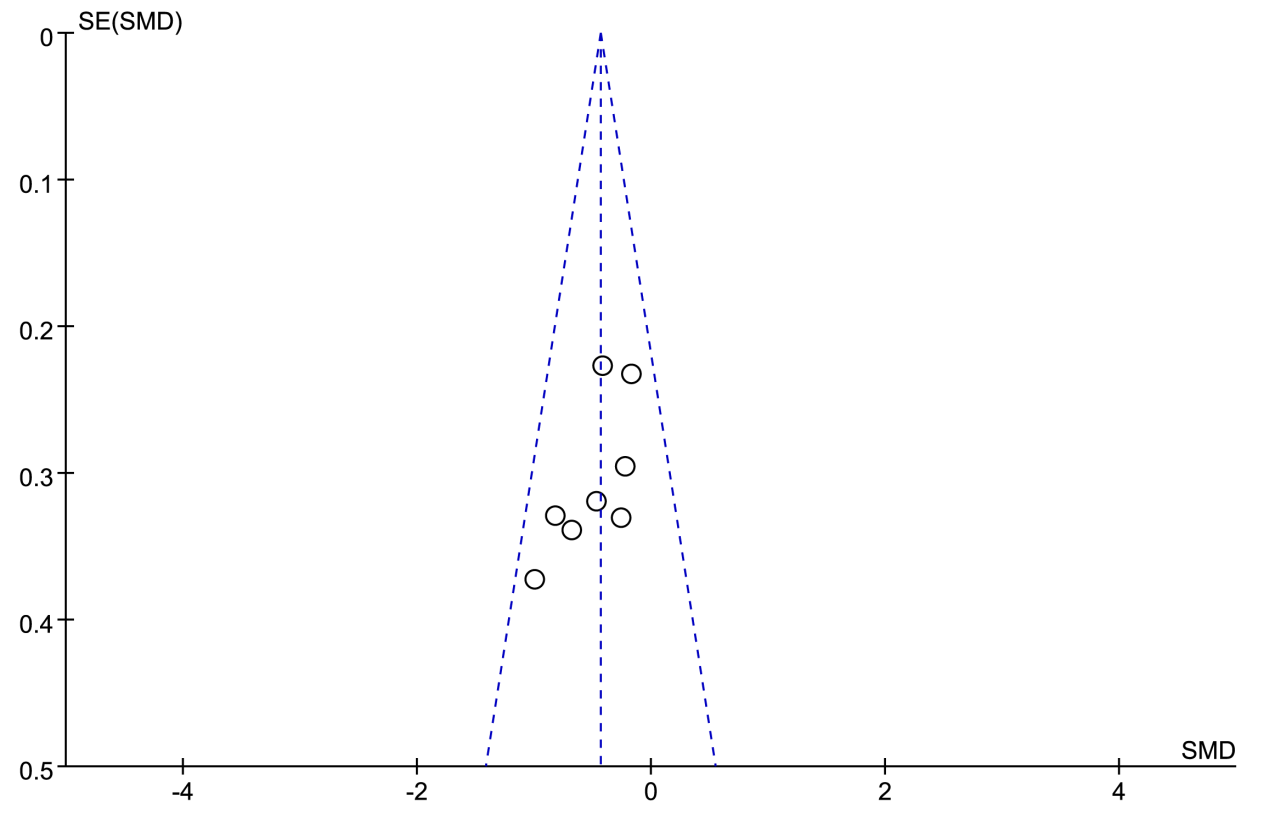
**

**Supplemental Figure 4** Forest for symptoms of re-experiencing

**
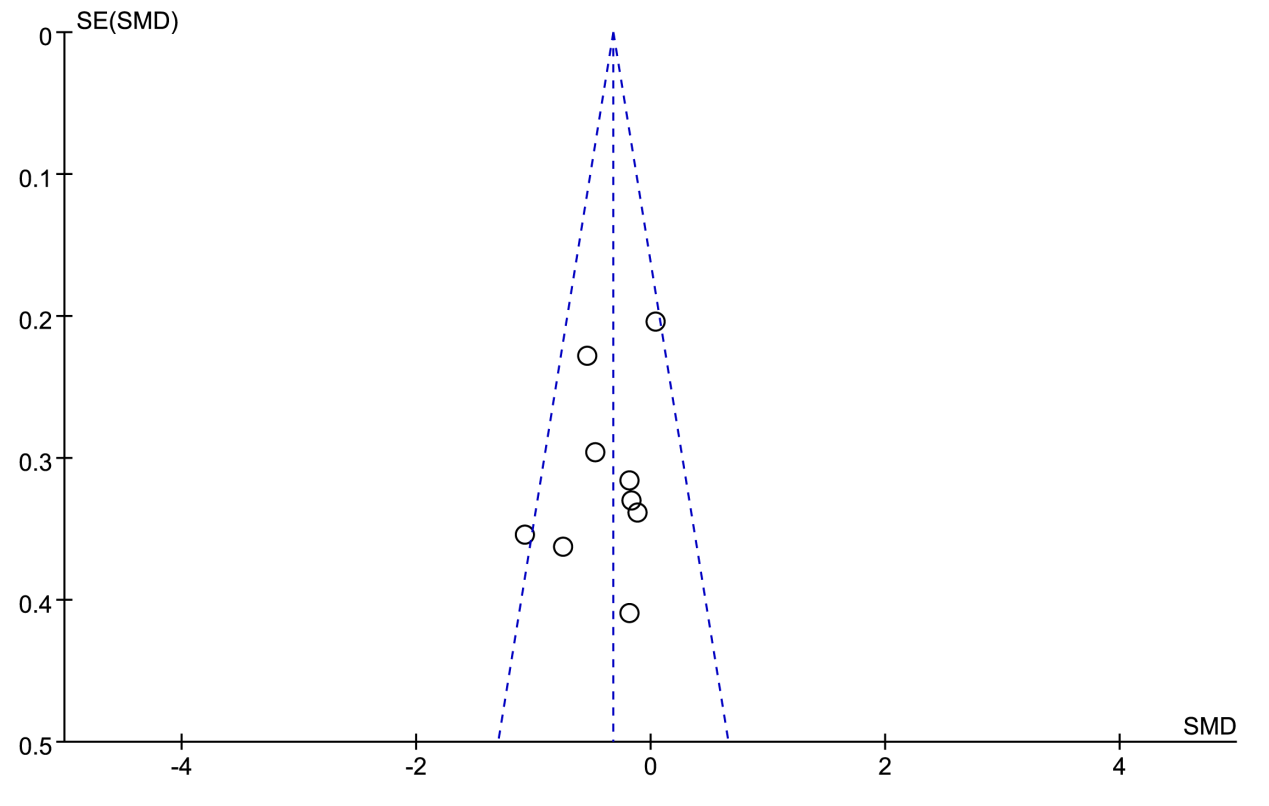
**

**Supplemental Figure 5** Forest for symptoms of avoidance

**
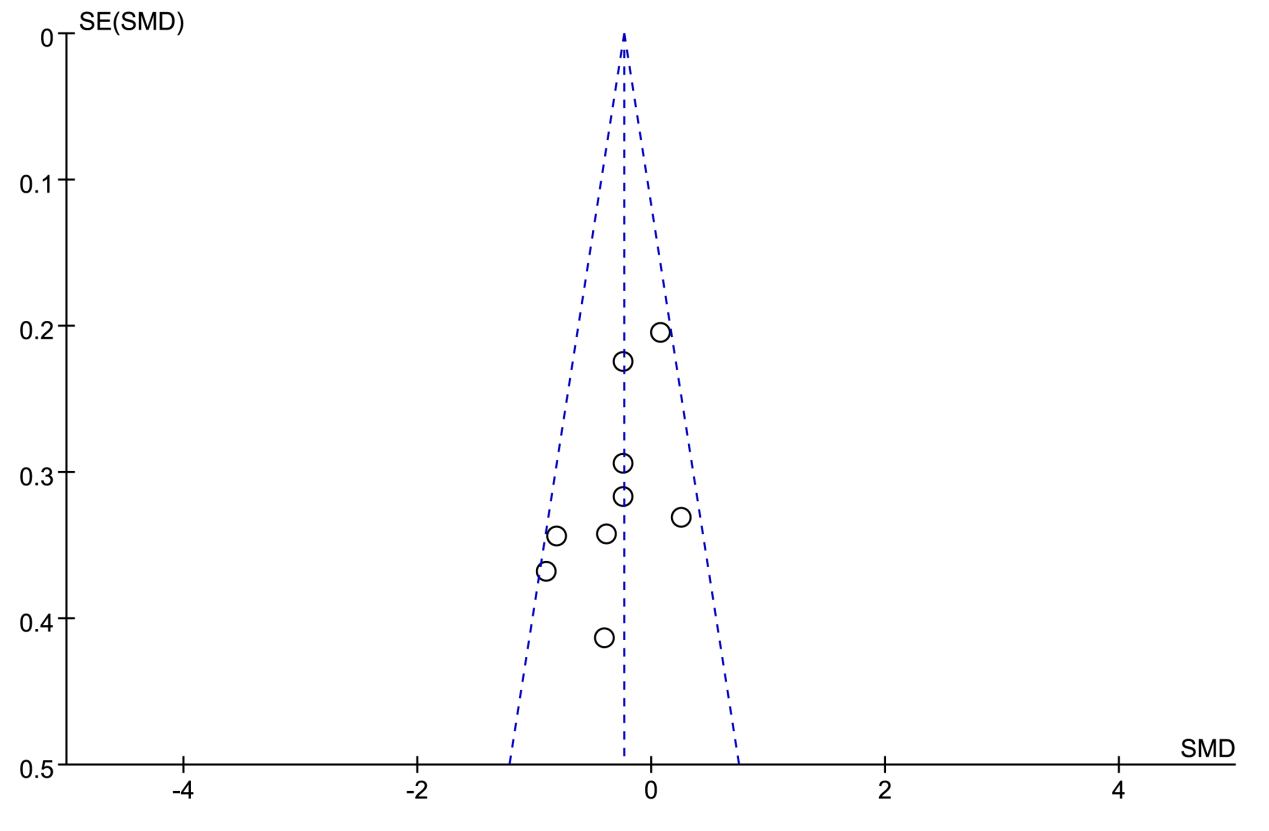
**

**Supplemental Figure 6** Forest for symptoms of hyper-arousal

**
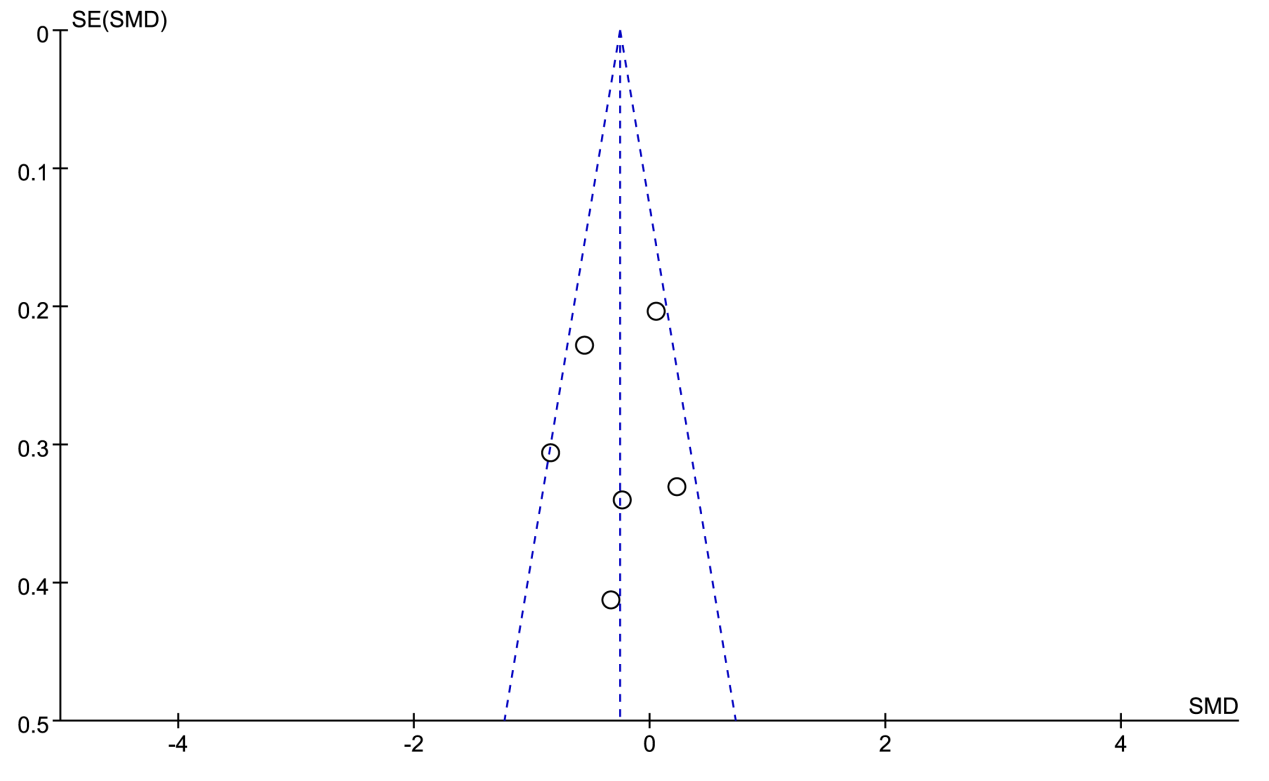
**

**Supplemental Figure 7** Forest for all-cause discontinuation rate

**
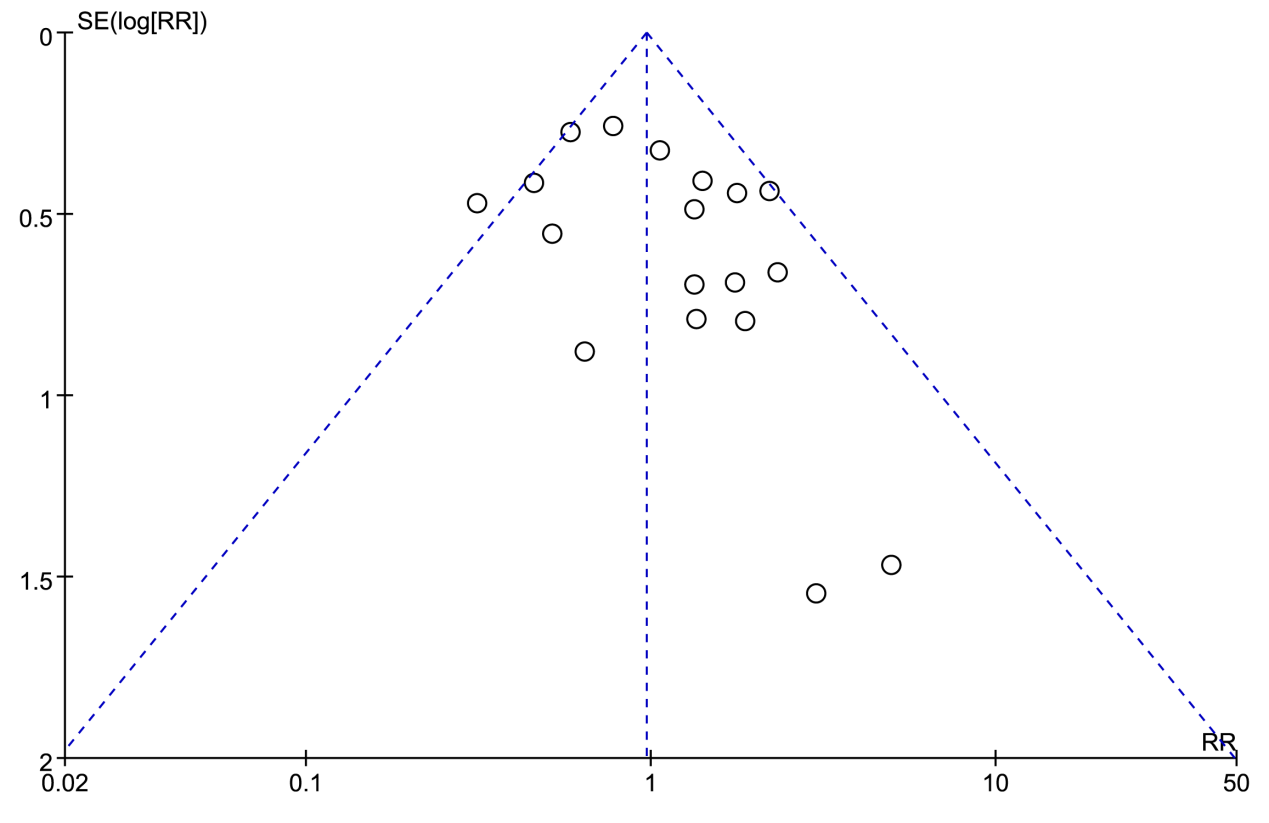
**

**Supplemental Figure 8** Forest for discontinuation rate due to AEs


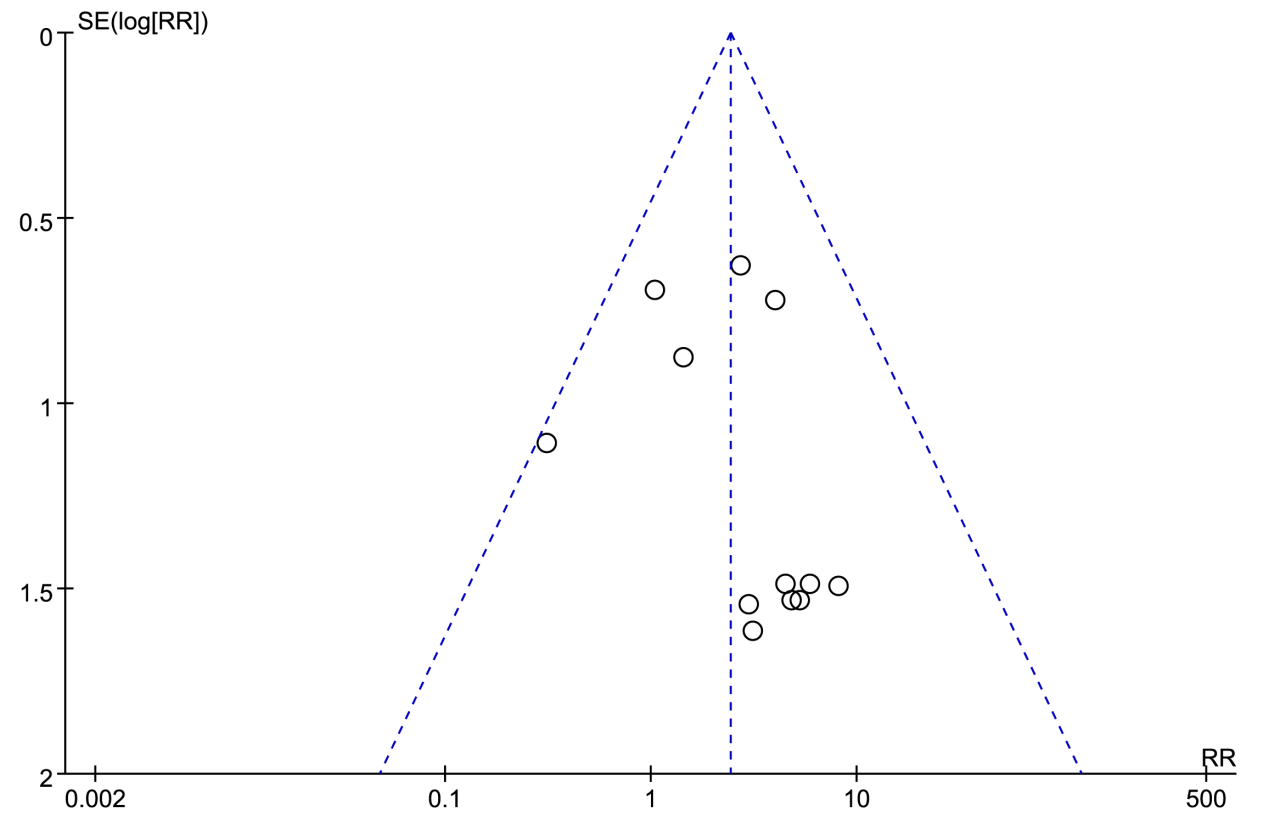

Supplement: Supplementary file 1 [file DataSheet1.docx]
